# Determination of correction factors in small MLC‐defined fields for the Razor and microSilicon diode detectors and evaluation of the suitability of the IAEA TRS‐483 protocol for multiple detectors

**DOI:** 10.1002/acm2.13657

**Published:** 2022-06-02

**Authors:** Andrew N. McGrath, Samane Golmakani, Timothy J. Williams

**Affiliations:** ^1^ W.P. Holman Clinic Royal Hobart Hospital Hobart Tasmania Australia

**Keywords:** diode, dosimetry, fff, radiation, small‐field

## Abstract

Small field output factors for Multileaf collimator (MLC)‐defined field sizes between 0.5 × 0.5 cm^2^ and 3 × 3 cm^2^ were measured with six different detectors for a Varian TrueBeam in 6‐MV, 6‐FFF, 10‐MV, and 10‐FFF photon beams. Correction factors $k_{Q_{\mathrm{clin}},Q_{\mathrm{ref}}}^{f_{\mathrm{clin}},f_{\mathrm{ref}}}$ from the IAEA publication TRS‐483 were used to correct the measured output factors. The corrected output factors from the six detectors were used to calculate correction factors for the PTW microSilicon T60023 (PTW, Freiburg, Germany) and IBA Razor (IBA Dosimetry, Schwarzenbruck, Germany) detectors. The uncertainty of the output and correction factors in this study was calculated and the calculations presented in detail. The application of the TRS‐483 correction factors significantly reduced the variation in output factors between the various detectors, with the exception of the PTW 60016 diode in 6‐MV and 6‐FFF beams, and the IBA PFD in 10‐MV and 10‐FFF beams. Correction factors calculated for the Razor agreed within 2.9% of existing literature for all energies, while the microSilicon correction factors agreed within 1.6% to the literature for 6‐MV beams. The uncertainty in the microSilicon and Razor correction factors was calculated to be less than 0.9% (*k* = 1). This study shows that TRS‐483 correction factors reduce the variation in output factors between the detectors used in this study and presents a suitable method for determining correction factors for detectors with unpublished values.

## 1 INTRODUCTION

Modern radiotherapy techniques require the collection of small field dosimetry data, as input to treatment planning systems and to verify the accuracy of treatment delivery. The measurement of relative output factors in small fields presents additional difficulties compared to measurements made in larger fields. Volume averaging, perturbation of photon fluence, and non‐uniform energy response means that careful selection and evaluation of detectors are required.^1^ The IAEA publication TRS‐483^2^ recommends the use of detector‐specific correction factors for measuring relative output factors. TRS‐483 provides correction factors for common detectors that have been derived from both theoretical and experimental work.

The PTW microSilicon T60023 (PTW, Freiburg, Germany) and IBA Razor (IBA Dosimetry, Schwarzenbruck, Germany) detectors are unshielded silicon solid state detectors designed for small field dosimetry. TRS‐483 has no published correction factors for these detectors.

The microSilicon detector was characterized by Schönfeld et al.^3^ and Akino et al.^4^ in 6‐MV photon fields and was found to be suitable for small field dosimetry. Small field correction factors have been determined for 6‐MV beams from experimental^3^, ^5^ and Monte Carlo^5^ methods, while to the best of our knowledge no publications have derived correction factors for 10‐MV beams.

The IBA Razor has been designed as a replacement for the IBA SFD diode. Its suitability for small field dosimetry and superiority to its predecessor IBA SFD have been investigated by Reggiori et al.^6^. Moreover, Liu et al.,^7^ Casar et al.,^8^ and Gul et al.^9^ have determined small MLC field correction factors for the Razor detector using experimental methods for both 6‐MV and 10‐MV photon energies. Other studies have assessed the Razor for measuring cone output factors or factors for the CyberKnife system.^7^, ^10^, ^11^

This study derives correction factors for the microSilicon and Razor detectors for 6‐MV and 10‐MV photon beams, with and without flattening filters for nominal field sizes range between 0.5×0.5 cm^2^ and 3×3 cm^2^. Six detectors suitable for small field dosimetry, with correction factors listed in TRS‐483, were used to determine these correction factors and an assessment of the uncertainty of this method is presented. In addition, the suitability of TRS‐483 correction factors was examined by assessing the reduction in variation of the measured output factors after the application of these factors.

## 2 METHODS

### 2.1 Measurements

Relative output factors were measured for the eight detectors listed in Table 1. Measurements were made for 6‐MV, 6‐FFF, 10‐MV, and 10‐FFF photon beams (see Table 2) for a Varian TrueBeam linear accelerator equipped with a Millenium‐120 MLC. Each measurement was made after aligning the detector to the center of the radiation field in a PTW BeamScan water tank. The effective measurement point of each detector was placed at the center of the radiation field at 100 cm SAD and at 10 cm depth in water. All detectors used in this study were irradiated with the smallest dimension of their sensitive volume parallel to the beam axis.

**TABLE 1:** Characteristics of the detectors used in this study

	**Measurement volume (mm^3^)**	**Sensitive area^a^**	**Sensitive area thickness (mm)**	**Shielded**
PTW 60023 (microSilicon)	0.03	Radius 0.75 mm	0.018	N
IBA Razor	0.006	Radius 0.3 mm	0.02	N
PTW 60019 (microDiamond)	0.004	Radius 1.1 mm	0.001	N
Sun Nuclear Edge	0.0019	l/w 0.8 mm	0.03	N
IBA SFD	0.017	Radius 0.3 mm	0.06	N
IBA EFD3G	0.19	Radius 1 mm	0.06	N
IBA PFD3G	0.19	Radius 1 mm	0.06	Y
PTW 60016	0.03	Radius 0.56 mm	0.03	Y

*Note*: ^a^The Sun Nuclear Edge detector has a square sensitive area, while all other detectors in this study are circular.

**TABLE 2:** Beam characteristics of the Varian TrueBeam linear accelerator used in this study, for a 10 × 10 cm^2^ reference field

**Beam**	**TPR_20,10_**	**PDD(10) (%)**
6‐MV	0.666	66.4
6‐FFF	0.630	63.2
10‐MV	0.738	73.5
10‐FFF	0.707	70.8

MLCs were used to collimate the radiation beam, with the accelerator jaws set to 0.5 cm behind each edge of the MLC field. The effective field sizes (*s*_eff_) of each MLC‐defined field were measured by the microSilicon detector at the depth of measurement and are listed in Table 3. The effective field size was calculated using the FWHM of the in‐plane (*y*) and cross‐plane (*x*) profiles with Equation (1):

(1) $$\;s_{\mathrm{eff}}=\sqrt{\mathrm{FHW}M_{x}\times \mathrm{FWH}M_{y}}.\;$$

The effective field sizes measured with the microSilicon detector were within 0.1 mm of the average *s*_eff_ measured by all other detectors in this study.

**TABLE 3:** The effective field *s*_eff_ (cm) of each MLC‐defined field used in this study

	Nominal square field size (cm)			
Energy	0.5	1.0	2.0	3.0
6‐MV	0.55	1.01	2.02	3.02
6‐FFF	0.51	1.01	2.00	2.99
10‐MV	0.59	1.07	2.04	3.03
10‐FFF	0.52	1.02	1.99	2.99

### 2.2 Calculation of relative output factors and derived correction factors

Output factors for the detectors listed in Table 1 (excluding microSilicon and Razor) were calculated using Equation (2) below, with the size of the machine‐specific reference field being 4×4 cm^2^:

(2) $$\Omega {}_{Q_{\mathrm{clin}},{Q_{4\times 4}}_{\det i}}^{f_{\mathrm{clin}},f_{4\times 4}}={\left[\frac{M_{Q_{\mathrm{clin}}}^{f_{\mathrm{clin}}}}{M_{Q_{4\times 4}}^{f_{4\times 4}}}\times \frac{k_{Q_{\mathrm{clin}},Q_{10\times 10}}^{f_{\mathrm{clin}},\;f_{10\times 10}}}{k_{Q_{4\times 4},Q_{10\times 10}}^{f_{4\times 4},f_{10\times 10}}}\right]}_{\det i}\;.$$

We have calculated correction factors $k_{Q_{\mathrm{clin}},Q_{4\times 4}}^{f_{\mathrm{clin}},f_{4\times 4}}$for the microSilicon and Razor detectors through the equation:

(3) $$\;k{}_{Q_{clin},{Q_{4\times 4}}_{\det x}}^{f_{clin},f_{4\times 4}}=\frac{\sum_{i=1}^{N}\Omega {{{}_{Q_{clin},Q_{4\times 4}}^{f_{clin},f_{4\times 4}}}_{\det i}}_{\;}}{N}\;\times \;{\left[\frac{M_{Q_{4\times 4}}^{f_{4\times 4}}}{M_{Q_{clin}}^{f_{clin}}}\right]}_{\det x},$$

where *M* is the electrometer reading, detx specifies either the microSilicon or Razor detector and deti specifies one of the detectors in this study that had published correction factors in TRS‐483.

### 2.3 Uncertainty budget

The relative standard uncertainty of the measurements was determined for each field size. Contributions to the total uncertainty include the output constancy of the linear accelerator, positioning error of the detector at the central axis (CAX), full width half maximum (FWHM) measurements and uncertainty in the correction factors taken from Tables 26 and 27 of TRS‐483.

***Output constancy***: The relative uncertainty due to the output constancy of the linear accelerator was measured. This was obtained using the standard deviation of repeated output measurements for a 4 × 4 cm^2^ field.

***CAX positioning***: According to the technical specification of the PTW BeamScan, detector positioning accuracy of the system is less than or equal to 0.1 mm. The percentage dose variations within 0.1 mm off‐axis were obtained from in‐plane and cross‐plane profiles of each field size for each energy. The maximum value of the variation in the profiles for each field size was used in the uncertainty calculation.

**Effect of FWHM on** $k_{Q_{\mathrm{clin}},\;Q_{\mathrm{msr}}}^{f_{\mathrm{clin}},f_{\mathrm{msr}}}$: The FWHM of each measured profile was used to interpolate correction factors $k_{Q_{\mathrm{clin}},\;Q_{\mathrm{msr}}}^{f_{\mathrm{clin}},f_{\mathrm{msr}}}$ from Tables 26 and 27 of TRS‐483. The uncertainty in determining FWHM was calculated from the standard deviation of the FWHM measurements for in‐plane and cross‐plane profiles using all detectors. The effect of the FWHM uncertainty on interpolating TRS‐483 correction factors was calculated for all detectors and energies and the maximum relative uncertainty obtained for each field size was used for the final uncertainty budget calculation.

$k_{\mathrm{TRS}-438}$: The relative uncertainties of the detector correction factors were taken from Table 37 of TRS‐483 for shielded and unshielded diodes/microDiamond.

The total relative standard uncertainty in measurement of field “s” was calculated by quadratic summation of the uncertainties with Equation (4) as follows:

(4) $$\;u_{m,s,i\;}=\sqrt{u_{\mathrm{output}}^{2}+u_{\mathrm{CAX},\;s}^{2}}\;.$$

The relative uncertainty of the correction factors $k_{Q_{\mathrm{clin}},\;Q_{\mathrm{msr}}}^{f_{\mathrm{clin}},f_{\mathrm{msr}}}$ was calculated based on Equation (5):

(5) $$\;u_{k,\;s,\;i\;}=\sqrt{u_{\mathrm{TRS}-483,s}^{2}+u_{\mathrm{FWHM},s}^{2}}\;.$$

To determine the relative standard uncertainty of the output factors measured by detector “*i*”, the total relative uncertainty calculated for each detector and field size was summed in quadrature with the total relative uncertainty of the 4×4 cm^2^ reference field calculated with Equation (6):

(6) $$\;u_{\Omega ,\;s,\;i\;}=\sqrt{u_{m,s,i}^{2}+u_{m,4\times 4,i}^{2}+u_{k,\;s,\;i}^{2}+u_{k,\;4\times 4,\;i}^{2}}\;.$$

To obtain the total relative uncertainty of the correction factors for the microSilicon and Razor detectors, the relative uncertainty of the average output factors was calculated using Equation (7), where it was assumed that the output factors measured by each detector were uncorrelated for simplicity:

(7) $$\;u_{\mathrm{Ave}\;\Omega }=\frac{\sqrt{\sum_{i=1}^{N}u_{\Omega ,\;s,\;i}^{2}}}{\sqrt{N}}\;,$$

where *N* is the number of detectors used to calculate the correction factors. For the 0.5 × 0.5 cm^2^ field size N=3 and for larger field sizes N=4or5. Then, $\;u_{Ave\;\Omega }$ was summated in quadrature with the microSilicon and Razor detectors uncertainties $u_{m,s,x}$ as shown in Equation (8):

(8) $$u_{\Omega ,\;s,\;x\;}=\sqrt{u_{Ave\;\Omega }^{2}+u_{m,\;s,\;x}^{2}+u_{m,\;4\times 4,\;x}^{2}}\;.$$

## 3 RESULTS

### 3.1 Relative output factors

**FIGURE 1:**
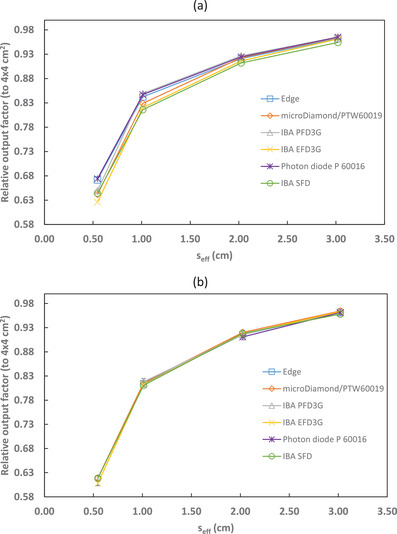
6‐MV output factors (relative to a 4 × 4 cm^2^ reference field) for several detectors with (a) uncorrected and (b) corrected by $k_{Q_{\mathrm{clin}},Q_{4\times 4}}^{f_{\mathrm{clin}},f_{4\times 4}}$

**FIGURE 2:**
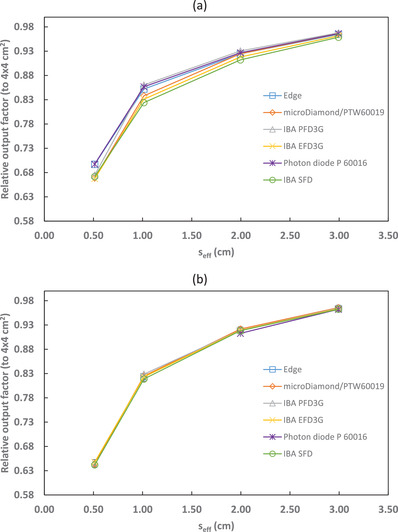
6‐FFF output factors (relative to a 4 × 4 cm^2^ reference field) for several detectors with (a) uncorrected and (b) corrected by $k_{Q_{\mathrm{clin}},Q_{4\times 4}}^{f_{\mathrm{clin}},f_{4\times 4}}$

**FIGURE 3:**
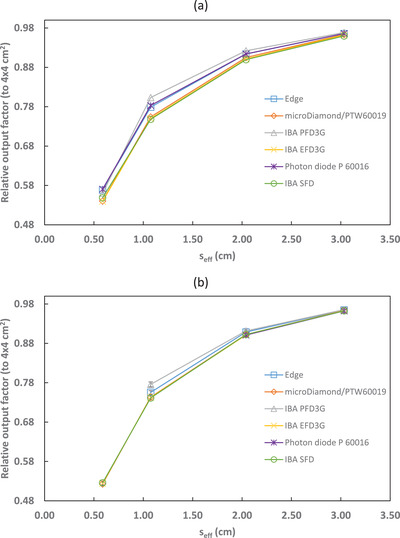
10‐MV output factors (relative to a 4 × 4 cm^2^ reference field) for several detectors with (a) uncorrected and (b) corrected by $k_{Q_{\mathrm{clin}},Q_{4\times 4}}^{f_{\mathrm{clin}},f_{4\times 4}}$

**FIGURE 4:**
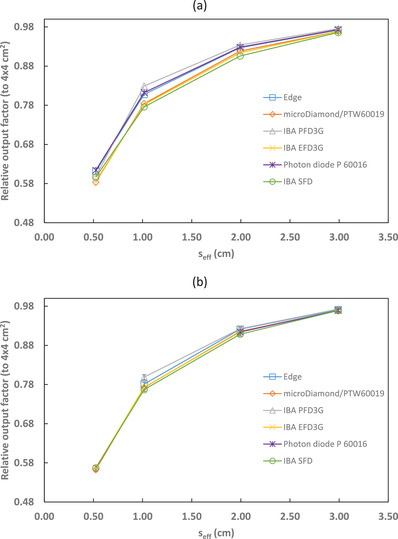
10‐FFF output factors (relative to a 4 × 4 cm^2^ reference field) for several detectors with (a) uncorrected and (b) corrected by $k_{Q_{\mathrm{clin}},Q_{4\times 4}}^{f_{\mathrm{clin}},f_{4\times 4}}$

Output factors relative to the 4 × 4 cm^2^ reference field, both corrected and uncorrected for $k_{Q_{\mathrm{clin}},Q_{4\times 4}}^{f_{\mathrm{clin}},f_{4\times 4}}$, are shown in Figures 1, 2, 3, 4 and Tables 4, 5, 6, 7. The uncertainties presented with the corrected output factors are k=1. As expected, the agreement between detectors improved when correction factors were applied. The PTW 60016 diode was excluded from calculating the 6‐MV and 6‐FFF calculation factors and uncertainties as it was identified as an outlier for the 2 × 2 cm^2^ field corrected output factors. Additionally, the IBA PFD detector showed a larger disagreement with the other detectors used in this study for 10‐MV and 10‐FFF beams and so was excluded from the calculation of the microSilicon and Razor correction factors and uncertainties. Empty cells in Tables 4 and 7 show where correction factor data were not available in TRS‐483. Extrapolation of correction factors was not performed to minimize uncertainty.

**TABLE 4:** Corrected output factors for 6‐MV. Cells marked with * show those that were not included in the correction factor calculation. Uncertainties (*k* = 1) are presented in parentheses

	***s*_eff_ (cm)**			
Detector	0.55	1.01	2.02	3.02
Edge	–	0.814 (0.005)	0.916 (0.005)	0.961 (0.005)
microDiamond/PTW60019	0.619 (0.005)	0.816 (0.005)	0.920 (0.005)	0.965 (0.005)
IBA PFD3G	–	0.818 (0.007)	0.916 (0.006)	0.962 (0.006)
IBA EFD3G	0.608 (0.005)	0.812 (0.005)	0.917 (0.005)	0.963 (0.005)
PTW 60016	–	–	0.911 (0.006)*	0.961 (0.006)*
IBA SFD	0.618 (0.005)	0.811 (0.005)	0.918 (0.005)	0.958 (0.005)

**TABLE 5:** Corrected output factors for 6‐FFF. Cells marked with * show those that were not included in the correction factor calculation. Uncertainties (*k* = 1) are presented in parentheses

	***s*_eff_ (cm)**			
Detector	0.55	1.01	2.02	3.02
Edge	–	0.824 (0.005)	0.919 (0.005)	0.964 (0.005)
microDiamond/PTW60019	0.644 (0.005)	0.825 (0.005)	0.922 (0.005)	0.966 (0.005)
IBA PFD3G	–	0.829 (0.007)	0.919 (0.006)	0.965 (0.006)
IBA EFD3G	0.647 (0.005)	0.823 (0.005)	0.919 (0.005)	0.964 (0.005)
PTW 60016	–	–	0.913 (0.006)*	0.962 (0.006)*
IBA SFD	0.642 (0.005)	0.819 (0.005)	0.918 (0.005)	0.963 (0.005)

**TABLE 6:** Corrected output factors for 10‐MV. Cells marked with * show those that were not included in the correction factor calculation. Uncertainties (*k* = 1) are presented in parentheses

	***s*_eff_ (cm)**			
Detector	0.59	1.07	2.04	3.03
Edge	–	0.755 (0.005)	0.909 (0.005)	0.965 (0.005)
microDiamond/PTW60019	0.523 (0.004)	0.744 (0.004)	0.902 (0.005)	0.964 (0.005)
IBA PFD3G	–	0.776 (0.006)*	0.912 (0.006)*	0.966 (0.006)*
IBA EFD3G	0.525 (0.004)	0.742 (0.004)	0.901 (0.005)	0.963 (0.005)
PTW 60016	–	–	0.901 (0.006)	0.962 (0.006)
IBA SFD	0.526 (0.004)	0.741 (0.004)	0.902 (0.005)	0.963 (0.005)

**TABLE 7:** Corrected output factors for 10‐FFF. Cells marked with * show those that were not included in the correction factor calculation. Uncertainties (*k* = 1) are presented in parentheses

	***s*_eff_ (cm)**			
	**0.52**	**1.02**	**1.99**	**2.99**
Edge	–	0.781 (0.005)	0.921 (0.005)	0.971 (0.005)
microDiamond/PTW60019	0.563 (0.005)	0.772 (0.005)	0.916 (0.005)	0.969 (0.005)
IBA PFD3G	–	0.799 (0.006)*	0.922 (0.006)*	0.973 (0.006)*
IBA EFD3G	0.567 (0.005)	0.773 (0.005)	0.914 (0.005)	0.969 (0.005)
PTW 60016	–	–	0.914 (0.006)	0.969 (0.006)
IBA SFD	0.567 (0.005)	0.767 (0.005)	0.909 (0.005)	0.969 (0.005)

### 3.2 Correction factors

The calculated correction factors $k_{Q_{\mathrm{clin}},Q_{4\times 4}}^{f_{\mathrm{clin}},f_{4\times 4}}$ for the microSilicon and Razor detectors are shown in Table 8.

**TABLE 8:** Correction factors for the microSilicon and Razor detectors for all energies in this study. Uncertainties (*k* = 1) are presented in parentheses

	**Nominal square field size (cm)**			
Energy/detector	0.50	1.0	2.0	3.0
6‐MV				
Razor	0.957 (0.008)	0.997 (0.007)	1.003 (0.006)	1.001 (0.006)
microSilicon	0.976 (0.009)	0.991 (0.007)	0.998 (0.006)	0.998 (0.006)
6‐FFF				
Razor	0.961 (0.008)	0.998 (0.007)	1.005 (0.006)	1.003 (0.006)
microSilicon	0.976 (0.009)	0.989 (0.007)	1.000 (0.006)	1.000 (0.006)
10‐MV				
Razor	0.967 (0.008)	0.999 (0.007)	1.003 (0.006)	1.001 (0.006)
microSilicon	0.994 (0.009)	0.999 (0.007)	1.002 (0.006)	1.002 (0.006)
10‐FFF				
Razor	0.963 (0.008)	0.995 (0.007)	1.003 (0.006)	1.002 (0.006)
microSilicon	0.986 (0.009)	0.993 (0.007)	0.999 (0.006)	1.001 (0.006)

**FIGURE 5:**
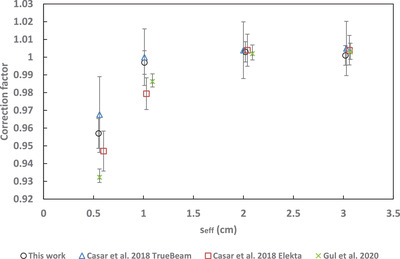
Correction factors for the Razor detector in this work compared to the literature for 6‐MV

**FIGURE 6:**
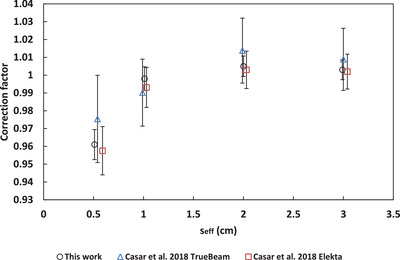
Correction factors for the Razor detector in this work compared to the literature for 6‐FFF

**FIGURE 7:**
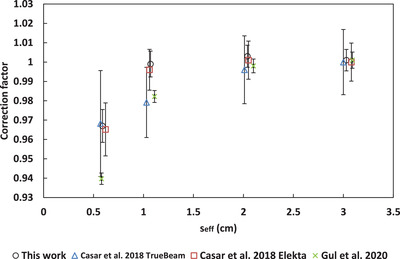
Correction factors for the Razor detector in this work compared to the literature for 10‐MV

**FIGURE 8:**
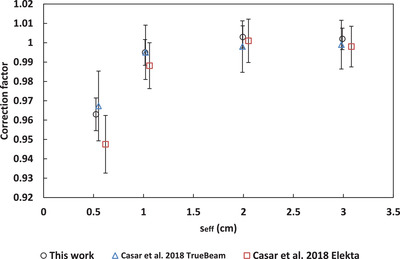
Correction factors for the Razor detector in this work compared to the literature for 10‐FFF

**FIGURE 9:**
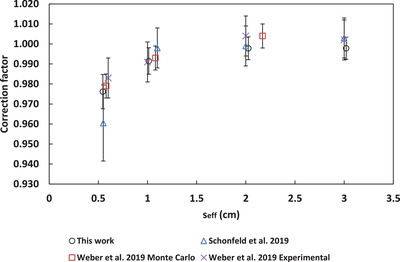
Correction factors for the microSilicon detector in this work compared to the literature for 6‐MV

A comparison to correction factors from the literature for the microSilicon^3^, ^5^ and Razor^8^, ^9^ is shown in Figures 5, 6, 7, 8, 9. No data for 6‐FFF, 10‐MV, and 10‐FFF was found in the literature to compare against microSilicon correction factors from this work.

### 3.3 Uncertainty budget

The relative standard uncertainty (*k* = 1) was calculated for the detector readings and the output factors, shown in Tables 9 and 10, respectively. Similarly, Table 11 shows the calculated uncertainties for the microSilicon and Razor correction factors.

**TABLE 9:** The relative standard uncertainty (%) in the measurement readings

	**Nominal square field size (cm)**				
Uncertainty	0.5	1.0	2.0	3.0	4.0
Output constancy (Type A)	0.03	0.03	0.03	0.03	0.03
CAX (Type A)	0.2	0.1	0.1	0.1	0.1
*K*_TRS‐483_ (Unshielded diodes/microDiamond) (Type B)	0.8	0.5	0.4	0.4	0.3
*K*_TRS‐483_ (Shielded diodes) (Type B)	1.3	0.7	0.5	0.5	0.4
Effect of FWHM on *K*_TRS‐483_ (Type A)	0.12	0.05	0.03	0.01	0.01
Total uncertainty (shielded)	–	0.7	0.5	0.4	0.4
Total uncertainty (unshielded diodes/microDiamond)	0.8	0.5	0.4	0.4	0.3

**TABLE 10:** The total relative standard uncertainty (%) for output factors

	**Nominal square field size (cm)**			
Detector type	0.5	1.0	2.0	3.0
Shielded diode	–	0.81	0.65	0.58
Unshielded diode/micro Diamond	0.85	0.60	0.52	0.52

**TABLE 11:** The total relative standard uncertainty for correction factors of both the microSilicon and Razor detectors

	**Nominal square field size (cm)**		
0.5	1	2	3
0.88	0.67	0.57	0.55

## 4 DISCUSSION

### 4.1 Relative output factors

The application of TRS‐483 correction factors reduced the variation in measured output factors between the six individual detectors in this study, with the exceptions of the PTW 60016 diode for 6‐MV and 6‐FFF beams and the IBA PFD3G for 10‐MV and 10‐FFF beams. This agrees with the work of Smith et al.^12^ who found that the PTW 60016 diode corrected output factors were lower than the average of other detectors for a 15 mm cone. It is not clear why the corrected 1×1 cm^2^ field measured with the IBA PFD3G showed a 4.2% and 3.2% difference to the average for 10‐MV and 10‐FFF beams respectively, however it may be notable that the correction factors listed in TRS‐483 are identical between 6‐MV and 10‐MV. Also, variation in detector construction and thus response may be responsible for the differences in corrected output factors. Finally, both the PTW 60016 and IBA PFD3G detectors are the only shielded diodes among the detectors studied. Shielded diodes exhibit greater perturbations than unshielded in small fields and have larger uncertainties in their correction factors.

The corrected output factors measured with the other five detectors agreed well. This follows the results of Smith et al.,^12^ who found that TRS‐483 correction factors reduced the variation in output factors for a variety of detectors. The application of TRS‐483 correction factors to small field measurements is a valid method to determine accurate output factors, however multiple detectors should be used to reduce the uncertainty of the results and limit the effect of variation amongst individual detectors.

### 4.2 Correction factors

Correction factors have been determined for 6‐MV, 6‐FFF, 10‐MV and 10‐FFF beams for the Razor and microSilicon detectors. The microSilicon correction factors for 6‐MV show a maximum difference of 1.6% for a 0.5 × 0.5 cm^2^ field size to Schönfeld et al.^3^ and less than 0.7% to all field sizes of Weber et al.^5^

The Razor correction factors showed a larger range of disagreement with the literature than the microSilicon. The largest difference was 2.9% to Gul et al.^9^ for a 0.5 × 0.5 cm^2^ field size. Our correction factors agreed more closely with Caser et al.,^8^ with agreement ranging between −1.5% and 2.0%. Additionally, Lui et al.^7^ published correction factors to a reference field of 3 × 3 cm^2^ for the Razor detector. When Equation (7) is modified to use a 3 × 3 cm^2^ reference field, our data agree with Liu within 2.0% for 6‐MV, 6‐FFF, 10‐MV, and 10‐FFF.

Calculation of correction factors for a new detector is often performed by Monte Carlo simulations or experimental work comparing to a “gold standard” detector, such as film or plastic scintillator. In this study, we have calculated correction factors using corrected output factors from 4–5 other detectors that have published correction factors. The variation in corrected output factors is small between the detectors and using 4–5 detectors results in an uncertainty of less than 0.9% (*k* = 1). Good agreement to published 6‐MV factors for the microSilicon suggests that this method is valid for 6‐FFF, 10‐MV, and 10‐FFF.

### 4.3 Uncertainty budget

The largest source of uncertainty in the calculated microSilicon and Razor factors is the correction factors taken from TRS‐483, which contribute between 0.3% and 0.75% for unshielded diodes/microDiamond and between 0.4% and 1.3% for shielded diodes. The second largest source of uncertainty is the detector positioning on the CAX which is calculated to be 0.2% for the 0.5 × 0.5 cm^2^ field and 0.1% for the other field sizes. This is smaller than the uncertainty calculated by Smith et al.^12^, which was between 0.6% and 0.3% for 1 × 1 cm^2^ to 4 × 4 cm^2^ field sizes for unshielded diodes/microDiamond and between 0.54% and 0.14% for shielded diodes. This may be due to the 0.3 mm shift used in their study to obtain the uncertainty due to CAX positioning, versus the 0.1 mm shift that was used in this study. On the other hand, our CAX uncertainty is larger than Tolabib et al.^13^ who calculated a significantly smaller uncertainty with values that were between 0.001 to 0.012% for a range of different detectors. Tolabib et al.^13^ do not specify the applied shift for calculating CAX uncertainty (μ_scan_) in their study and so it is not clear from where this difference arises.

The uncertainties due to output constancy and effect of FWHM on K_TRS‐483_ are small compared to the other uncertainties discussed. The total relative standard uncertainty for detector measurements was similar to the values found by Smith et al.^12^, with a maximum difference of 0.3% for 1 × 1 cm^2^ field size for unshielded diodes/microDiamond. It should be noted that Smith et al.^12^ did not include any data for 0.5 × 0.5 cm^2^ square fields.

The relative standard uncertainty calculated for the correction factors of microSilicon and Razor in this study are close to (within 0.3%) the uncertainty calculated for the unshielded diodes/microDiamond in Table 26 of TRS‐483 across the measured field sizes.

## 5 CONCLUSION

TRS‐483 correction factors were found to reduce the variation in measured small‐field output factors for six detectors. Five of those detectors were used to calculate correction factors for the microSilicon and Razor detectors, which agreed well with factors in the literature. The approach taken in this study, which was to calculate correction factors from the average of a number of corrected detector measurements, was shown to result in an acceptable level of uncertainty.

## AUTHORS’ CONTRIBUTION

Andrew N. McGrath and Samane Golmakani wrote the manuscript and collected outcome data. Samane Golmakani calculated and wrote the uncertainty budget sections. Timothy J. Williams helped develop the technique and provided proofreading for the manuscript.

## CONFLICT OF INTEREST

The authors declare that there is no conflict of interest that could be perceived as prejudicing the impartiality of the research reported.

## Supporting information




Supplementary information
