# Supplementary material for: Determination of correction factors in small MLC‐defined fields for the Razor and microSilicon diode detectors and evaluation of the suitability of the IAEA TRS‐483 protocol for multiple detectors
Source: J Appl Clin Med Phys. 2022 Jun 2;23(7):e13657. doi: 10.1002/acm2.13657 (PMC9278669; doi:10.1002/acm2.13657)
Supplement: Supplementary file 1 — Supplementary information [file ACM2-23-e13657-s001.docx]

**Determination of correction factors in small MLC-defined fields for the Razor and microSilicon diode detectors and evaluation of the suitability of the IAEA TRS-483 protocol for multiple detectors**

Andrew N. McGrath

Radiation Oncology Medical Physicist

W.P Holman Clinic

Royal Hobart Hospital,

38 Liverpool Street,

Hobart, TAS, 7009

andrew.n.mcgrath@gmail.com

**Suggested running title:** Small field factors for diodes.

Samane Golmakani

Radiation Oncology Medical Physicist

W.P Holman Clinic

Royal Hobart Hospital,

38 Liverpool Street,

Hobart, TAS, 7009

Timothy J. Williams

Deputy Chief Radiation Oncology Medical Physicist

W.P Holman Clinic

Royal Hobart Hospital,

38 Liverpool Street,

Hobart, TAS, 7009

**Author Contribution Statement**

Andrew N. McGrath and Samane Golmakani wrote the manuscript and collected outcome data. Samane Golmakani calculated and wrote the uncertainty budget sections. Timothy J. Williams helped develop the technique and provided proofreading for the manuscript.

**Acknowledgements**

The authors would like to thank all Radiation Oncology staff in the Tasmanian Health Service for their support with this work.

**Keywords**

Small-field, dosimetry, radiation, diode, fff

**Corresponding author:**

Andrew N. McGrath

Radiation Oncology Medical Physicist

W.P Holman Clinic

Royal Hobart Hospital,

38 Liverpool Street,

Hobart, TAS, 7009

andrew.n.mcgrath@gmail.com
